# Integrated Airborne LiDAR Data and Imagery for Suburban Land Cover Classification Using Machine Learning Methods

**DOI:** 10.3390/s19091996

**Published:** 2019-04-28

**Authors:** You Mo, Ruofei Zhong, Haili Sun, Qiong Wu, Liming Du, Yuxin Geng, Shisong Cao

**Affiliations:** 1 Beijing Advanced Innovation Center for Imaging Theory and Technology, Key Lab of 3D Information Acquisition and Application, MOE, Capital Normal University, Beijing 100048, China; 2173601004@cnu.edu.cn (Y.M.); sunhaili@cnu.edu.cn (H.S.); 2150901014@cnu.edu.cn (Q.W.); 2160901010@cnu.edu.cn (L.D.); 2180901014@cnu.edu.cn (Y.G.); shisongcao@cnu.edu.cn (S.C.); 2 Beijing Key Laboratory for Geographic Information Systems and Environment and Resources, Capital Normal University, Beijing 100048, China

**Keywords:** suburban land cover classification, LiDAR, aerial imagery, machine learning, nDSM, surface roughness, surface intensity

## Abstract

It is valuable to study the land use/land cover (LULC) classification for suburbs. The fusion of Light Detection and Ranging (LiDAR) data and aerial imagery is often regarded as an effective method for the LULC classification; however, more in-depth analysis would be required to explore effective information for enhancing the suburban LULC classification. In this study, first, both aerial imageries and point clouds were simultaneously collected. Then, LiDAR-derived models, i.e., normalized digital surface model (nDSM) and surface intensity model (IM), were generated from the elevation and intensity of point clouds. Further, considering the surface characteristics of ground objects in suburb, we proposed a new LiDAR-derived model, namely surface roughness model (RM), to reveal the degree of surface fluctuations. Additionally, various combinations of aerial imageries and the LiDAR-derived data were used to analyze the effects of multi-variable fusion under different scenarios and optimize the multi-variable integration for suburban LULC classification. The mean decrease impurity method was used to identify the importance of variables; three machine learning classifiers, i.e., random forest (RF), k-nearest neighbor (KNN) and artificial neural network (ANN) were adopted in various scenarios. The results were as follows. The fusion of aerial imagery and all the LiDAR-derived models, i.e., nDSM, RM and IM, with RF classifier performs best in the suburban LULC classification (overall accuracy = 84.75%, kappa coefficient = 0.80). Variable importance analysis shows that nDSM has the highest variable importance proportion (VIP) value, followed by RM, IM, and spectral information, indicating the feasibility of this proposed LiDAR-derived model-RM. This research presents effective methods relating to the application of aerial imagery and LiDAR-derived model for the complex suburban surface scenarios.

## 1. Introduction

Land use/land cover (LULC) has significant influences on the biochemical cycle, hydrological processes, climate change, and regional ecosystem biodiversity from global to local scales [1,2]. Suburb is often regarded as an indispensable part of urban area, which undertakes the agricultural serving function of providing agricultural and sideline products for the city. With the acceleration of urbanization, the structure and management concerning the suburban LULC have undergone tremendous changes. Therefore, monitoring changes in suburban LULC classification are beneficial for exploring sustainable development of suburbs.

In recent years, remote sensing technology has developed into multi-platform, multi-sensor, and multi-angle featuring high spatial resolution, high spectral resolution, and high time-phase resolution, which are conducive to better interpretation and processing of images by users [2,3]. Laser scanners can accurately acquire the three-dimensional information (XYZ) of the Earth’s landscape and bare surface without the influence of illumination conditions, but the LiDAR data lack sufficient spectral information [4]. Since aerial imageries can provide spectral information in the visible band, the fusion of aerial imagery and LiDAR data can enable improvements in the classification accuracy of ground objects and make possible land cover surveys and applications at very fine scales [2,5,6,7,8].

Previous research has proven that the LiDAR-derived data, e.g., nDSM, intensity, and topographic information, could perform better in distinguishing ground objects [2,7,8,9]. For example, the nDSM enables the distinguishing of high vegetation (trees) from low vegetation according to their elevation differences, whereas the intensity could enable better distinguishing of different materials, especially in the classification of land cover. Nevertheless, the high volume of information contained in LiDAR data is of significant for further improving the accuracy of land cover classification. Some derived height features like skewness and kurtosis models [6], the height variation [10], mean, variance and standard derivation of height of the first echo [11], homogeneity, contrast, and entropy of height [12] were measured for improving the accuracy of classification, but these derived features did not perform any better than nDSM and intensity [2]. Therefore, some new feature descriptions of LiDAR data need to be discovered and verified to play a role in land cover classification. In addition, a literature review shows that most of the studies concerning urban land cover classification [2,5,13,14,15] were performed, however, few studies focused on the suburbs’ classification.

In the article, taking suburbs as the study area, the main objectives of the study are as follows:

(1) Analyze the performances of fusion data of LiDAR-derived models and aerial imageries incorporated with three machine learning classifiers (i.e., random forest (RF), k-nearest neighbor (KNN), and artificial neural network (ANN) [16,17,18,19,20]) for the suburban land cover classification.
(2) Propose a new index, namely surface roughness model (RM), to describe and quantify the surface fluctuations of ground objects, and further evaluate the effects of RM to improve the land cover classification.
(3) Calculate the contribution of various input variables, i.e., RGB, nDSM, RM, and IM, in the various scenarios, and determine the optimal combination scheme as well as the optimal classifier.

## 2. Materials and Methods

### 2.1. Study Area

**Figure 1:**
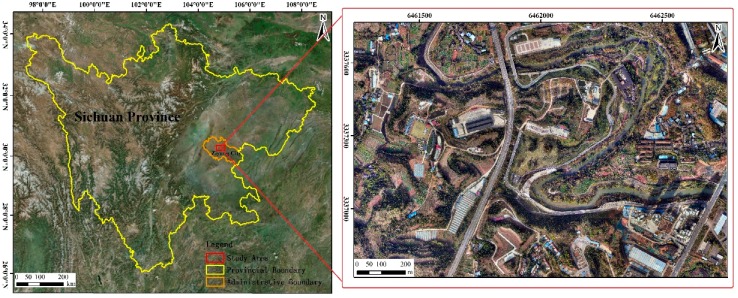
Location of study area.

Figure 1 gives an overview of the study area, which is located in the central part of Sichuan Basin and belongs to Ziyang City, Sichuan Province, China (104°59′ E, 30°14′ N). As a suburban region, the area features diverse and irregular land cover, and includes both natural and human formed landscapes, i.e., low buildings, roads, cultivated land, and vegetation. The study area is approximately 1.6 km² (1538 m × 1060 m).

### 2.2. Data Sources

Airborne LiDAR data and aerial imageries were collected in December 2017 using an integrated RIEGL VUX-1LR laser scanner recording a single pulse return and intensity, a PHASE ONE IXU1000-R digital camera, and the POS (Positioning and Orientation System). Additionally, the system acquires point clouds and aerial imageries synchronously. The parameters of those data were detailed as follows.

(1) Point clouds parameters: the density of the point clouds was approximately 20 points/m^2^. The horizontal and vertical resolution of the point clouds were approximately 0.17 m and 0.2 m, respectively. The maximum difference in altitude among the points was 138.94 m.
(2) Aerial imagery parameters: the image size was 11,608 × 8708 pixels. The visible bands included red, green and blue.

### 2.3. Methods

#### 2.3.1. Research Scheme Description

**Figure 2:**
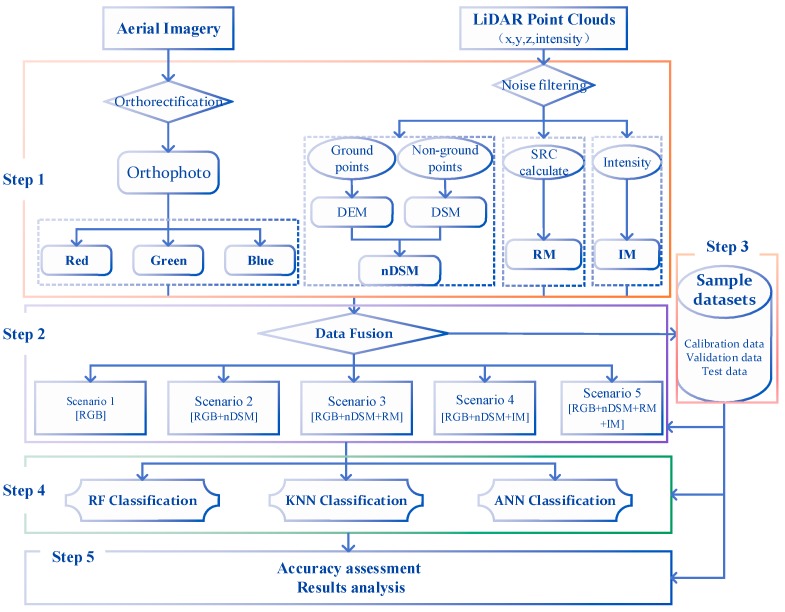
Study workflow.

The study workflow is shown in Figure 2 and included five key steps: (1) Data pre-processing, which included the processing of point cloud and aerial imageries; (2) Data fusion schemes design, which comprised five scenarios of data fusion for the point clouds and aerial imageries; (3) Sample datasets selection, which included training data collection, calibration and validation data division, and test data preparation; (4) Classifier selection, which used three machine learning algorithms included RF, KNN, and ANN; (5) Accuracy assessment and results analysis. 

#### 2.3.2. Data Pre-processing

(1) LiDAR-derived models

Three LiDAR-derived surface models, i.e., normalized digital surface model (nDSM), surface intensity model (IM) and surface roughness model (RM), were generated for the land cover classification.

Digital elevation model (DEM) describes ground elevation information, whereas digital surface model (DSM) reflects the realistic surface fluctuation [2]. nDSM is generated to eliminate the influence of topography and to obtain the accurate relative elevation differences between objects and the ground surface. Previous researchers have been demonstrated that the nDSM can be used as an effective indicator for improving classification accuracy [21,22]. The procedure of nDSM generation were as follows: Point cloud data were initially filtered to eliminate low points, air points and isolated points and then classified into ground, non-ground using Terrasolid software; then, DEM and DSM with a resolutions of 0.5 m covering the study area were established by the inverse distance weighted (IDW) interpolation algorithm using ground points and non-ground points, respectively; lastly, nDSM with a resolution of 0.5 m was generated by subtracting DSM and DEM. Specifically, we also performed some appropriate adjustments to ensure the accuracy of the nDSM (i.e., we set a conditional function to remove some negative values caused by laser penetration of glass roofs) [4].

For a discrete return LiDAR sensor, intensity describes the peak amplitude of the laser beam returned by the object [2], and distinguishes ground objects according to reflectance properties of objects [5,12,21,23]. The LiDAR data recorded the returns with 16 bits of high-resolution intensity information. In this study, intensity is regarded as one of the input parameters. IM with a resolution of 0.5 m was generated by the IDW algorithm using all points.

**Figure 3:**
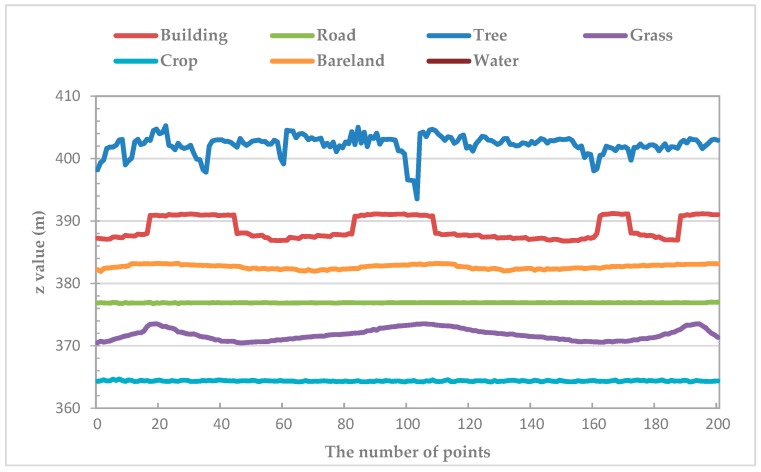
Profile of surface elevation fluctuation for seven classes.

The discrimination of many types of ground objects could depend on the elevation differences, e.g., trees and roads [2]. However, trees and low buildings feature similar elevations in some cases, especially in suburban areas. Figure 3 shows the profile of surface elevation fluctuations for various land covers. As shown in Figure 3, a significant difference in surface fluctuations was observed among land covers. It is clear from Figure 3 that trees had the highest surface fluctuations, whereas roads had the lowest surface fluctuations; crops and grasses had varying degrees of fluctuations; the surface fluctuations of buildings are low. Note that water is missing in Figure 3 because there are no point clouds in the water surfaces. In order to capture those differences of surface fluctuations among land covers, we defined a variable to describe and quantify the surface fluctuations of the ground objects.

Considering that the density of point clouds are sufficient, the surface roughness coefficient (SRC) were proposed to reflect the surface fluctuations of ground objects, and is defined as a ratio of the ground objects surface area and its projected area in a grid unit:

(1) $$SRC_{ij}=\frac{S_{sij}}{S_{pij}},$$

where $SRC_{ij}$ represents the roughness coefficient for the grid at row *i* and column *j*; $S_{sij}$ and $S_{pij}$ represent the surface area and projected area, respectively, in the grid at row *i* and column *j*.

**Figure 4:**
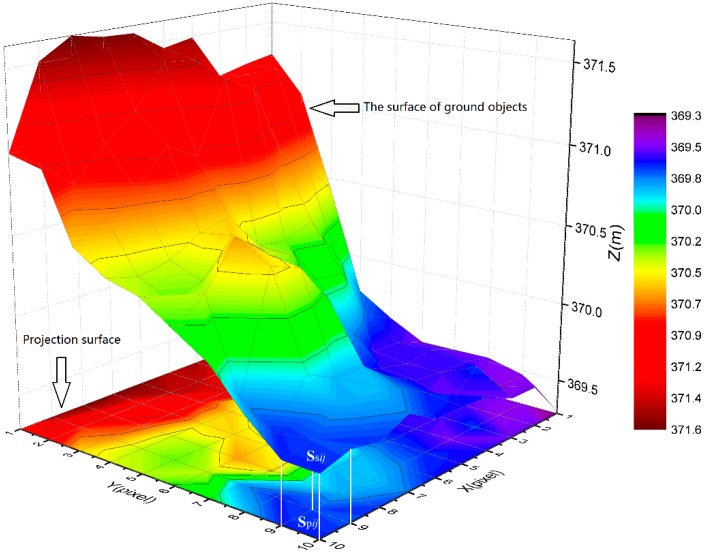
A three-dimensional display of the ground objects surface area of a grid unit and its projected area on the horizontal plane.

Figure 4 shows the ground objects surface and its projected surface. Firstly, we defined the size of the grid as 0.5 m × 0.5 m, and assigned corresponding points onto it. Then, we built polygons based on points within each grid. Next, we calculated the surface area of polygons within each grid, and found its corresponding horizontal projection region based on the position of the four vertices and calculated projection area, SRC was calculated by formula (1) until all grids are completed. Finally, IDW interpolation method was used to generate the RM with 0.5 m resolution by SRC.

**Figure 5:**
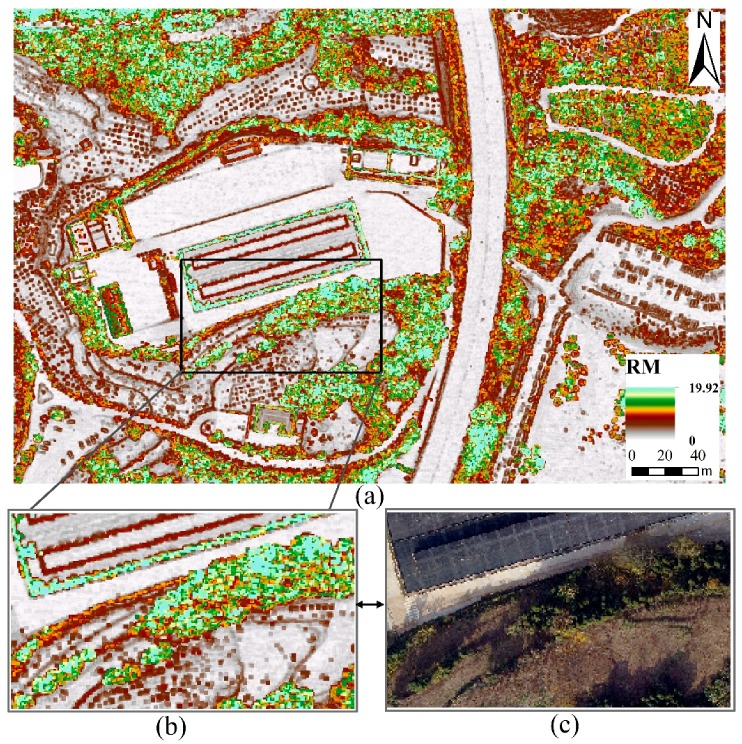
Spatial map of surface roughness model (RM): (**a**) RM; (**b**) a subgraph of (**a**); (**c**) the aerial imagery corresponding to the position of (**b**).

The results of RM are described in Figure 5, where RM in the water region is set to 0 because there are no point cloud data. As show in Figure 5a, the outline of the ground objects is distinct, and we can distinguish the classes clearly according to RM. Compared RM (Figure 5b) with its corresponding aerial imageries (Figure 5c), we found that the RM values for trees were the highest, crops and grasses were relatively low, and buildings and roads were the lowest. A comprehensive analysis Figure 3 and Figure 5, revealed that the roughness of trees is large when the elevation fluctuations are large, while the roughness of buildings and roads is small when the fluctuations are small. Therefore, RM as an overall feature of measuring the elevation variation of ground objects in a certain region, it reflects and quantifies the surface fluctuations degree of ground objects. We used RM as a new LiDAR-derived model and studied its effects and advantages in LULC classification in suburbs.

(2) Aerial imagery processing

There were 70 aerial images that covered the study area, and selected to generate orthophoto. Their position and orientation information were obtained from POS data and used in Pix4Dmapper software to generate the orthophoto. Firstly, we selected UTM Zone 48N/WGS 84 as the coordinate system and imported the position and orientation information of images. Secondly, we selected original image size to extract the image features and match the images. Thirdly, we optimized externals and all internals of images to rematch the images. Next, we used weighted average method to generate a complete orthophoto mosaic of study area. Finally, the orthophoto was sampled to 0.5 m resolution to maintain the resolution consistent with LiDAR-derived models.

#### 2.3.3. Data Fusion Schemes Design

This study was designed with five groups of data fusion scenarios. We fused the spectral information of aerial imagery and three LiDAR-derived models to analyze and assess differences in classification accuracy. We used an image layer stacking process to combine those data sources and generated the composite images. Table 1 shows the fused input bands used in scenarios.

#### 2.3.4. Classification Sample Datasets

**Figure 6:**
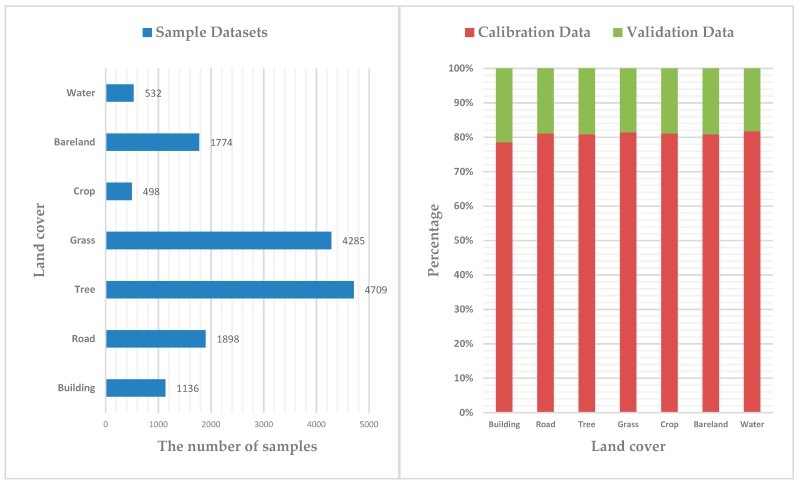
Sample datasets statistics of seven classes (**left**). The proportion of calibration and validation sample data in the sample datasets (**right**).

Based on the spectral response of features on the aerial images and field observation, seven land cover types, including building, road, tree, grass, crop, bareland, and water were identified. Samples covering the study area were extracted directly from the aerial images. Further, those samples were divided into calibration, validation and test samples. In order to avoid the influence of an imbalance of samples in quantity and proportion on classifier training [24], we used random sampling to create sample datasets overlaying the entire study area and all classes, the number of samples in each category was selected according to the proportion of categories in the entire study area. In the process of samples selection, we randomly created samples first, which did not contain any band information. Then, we extracted the band information from composite images, and assigned it to corresponding samples. The samples were divided into seven land covers by referring to aerial true color imageries. In addition, some poor-quality training samples were removed in the process of sample classification. Again, we used the random sampling to ensure the representative class proportions and minimize the proportional error in the training results, where 80% of the classified samples were selected as the calibration datasets to train the classifier, and the remaining 20% as the validation datasets to verify the accuracy of the classification model (Figure 6). Meanwhile, we used the data of the entire research area which contained different band combination information as the test dataset to output the classification map. All the sample datasets were converted to decimal data format.

#### 2.3.5. Classifier Selection

Three machine learning methods were adopted to perform the land cover classification and identify the consistency of the classification results, included RF, KNN and ANN. The algorithms were implemented in the Python 3.6 environment.

RF is an important non-parametric ensemble learning method based on decision trees and bagging [25,26,27]. RF can handle thousands of input variables and evaluate the importance of variables. It can also maintain high classification accuracy even for a smaller number of training data [14,28,29]. The samples from the original training dataset are taken at random but with replacement using the bootstrap method to construct the decision tree. The Gini criterion is used to split each node [30]. The remaining original samples which are called out-of-bag (OOB) are used to estimate the test accuracy and evaluate the variables importance by measuring the mean decrease impurity [30]. OOB improves the efficiency and accuracy of classifier by optimizing parameters. Classification of new data by combining all the classification voting results of each constructed tree and assigns the class with the most votes as the final output [12,21]. The vital parameters for RF: the number of constructed decision trees (ntree); the number of randomly variables selected (mtree) used for the best split at each node. Some studies have indicated that ntree is more crucial than mtree to the classification accuracy [28,31], the default value of mtree is usually set to the square root of the number of input variables [32], while the ntree values ranging from 0 to 1000 have been shown to be effective for many RF applications [16,33,34]. Our research used the default value of mtree; set the ntree values range from 0 to 1000 at an interval of 100 to test the best value of ntree so as to get the optimal accuracy for the best classification of scenarios.

KNN algorithm [35,36] is a lazy learning algorithm (i.e., instance-based) and a powerful non-parametric classification method. The KNN does not require generating classifier in advance, it stores all available cases and classifies new cases based on a similarity measure which is the distance between different eigenvalues [37]. There are four main steps for KNN classification: assign a classification code to each data in the sample dataset; input the new data and compared its features with the sample dataset; extract the classification code according to the most similar feature (nearest neighbor) in the sample dataset; new data (a new instance) is classified by a majority vote of its neighbors, by assigning to the most common class among its k nearest neighbors (measured by a distance function; usually k < 20) [19,38]. The choice of k value is crucial in classification process, whether the value of k is large or small will lead to either overly normalize the patterns or highlight local variation problems [39,40]. Our research attempted to get optimal k for the best classification, by setting the value range from 0 to 20; then, an iterative algorithm was introduced to calculate the classification accuracy of each iteration; the k corresponding to the highest accuracy is selected as the optimal value which is determined by cross-validation using training data.

ANN algorithm is a mathematical model of distributed parallel information processing that imitates the behavior characteristics of biological neural network [41]. The neural networks get a solution using a non-algorithmic and unstructured fashion, also it depends on the complexity of the system to process information by adjusting the weight of a large number of internal nodes (neurons) which are connected to each other in the networks [42]. ANN has been used to classify various types of remote data and have in certain instances produced results superior to those of traditional statistical methods [20,43,44]. When using ANN to process remote sensing images, it could effectively reduce errors caused by human participation because the procedure basically controlled by program; additionally, the requirements of data are not constrained by normal distribution, and it can adaptively simulate complex and nonlinear patterns and give appropriate topologies. Our research chose a multilayer perceptron (MLP) as a classifier which is a kind of forward structure ANN model based on supervised training [20] to train the sample datasets.

#### 2.3.6. Accuracy Assessment

Accuracy assessment is a key step for the land cover classification. The classification accuracy directly affects the mapping report, and this report is a key reference for the usefulness of actual land cover data analysis and the rationality of scientific research. In addition, it is very important to understand and analyze the causes and distribution characteristics of classification errors to revise the classified results and improve the subsequent classification methods. 

Accuracy assessment is carried out by the confusion matrix (CM) [45], which is widely used for accuracy assessment [2,14], and describes the classification accuracy of classifiers. Moreover, CM can clearly get the number of correct classification and misclassified of each feature and category. The assessment indexes of CM include the overall accuracy (OA), kappa coefficient, user’s accuracy (UA) and producer’s accuracy (PA). The OA is a statistic with probabilistic significance, which represents the probability that all classification results are consistent with the actual types of the corresponding regions on the ground for each random sample. The kappa coefficient analysis is an objective evaluation index, which adopts a discrete multivariate technology and takes into account all factors of the matrix. The UA measures commission error, and represents how well a pixel classified as a certain land cover type matches the actual ground type. The PA measures omission error, and represents how well a land cover class can be assigned to the landscape. [45,46]. CM is an n×n matrix, the OA, UA, PA, and kappa coefficient can be defined as:

(2) $$\mathrm{OA}=\sum_{k=1}^{n}p_{kk}/p,$$

(3) $$\mathrm{UA}=p_{ii}/p_{u},$$

(4) $$\mathrm{PA}=p_{jj}/p_{p},$$

(5) $$\mathrm{kappa}\;\mathrm{coefficient}=\frac{p\sum_{i=1}^{n}p_{ii}-\sum_{i=1}^{n}\left(p_{u}p_{u}\right)}{p^{2}-\sum_{i=1}^{n}\left(p_{u}p_{u}\right)}$$

where n is the total of classes, p is the total of samples, row i represents classified samples, column *j* represents practical samples, $p_{kk}$ represents the total number of *k*th class correctly classified, $p_{u}$ represents the sum of *i*th class from classification, $p_{p}$ represents the sum of *j*th class from practical samples.

**Figure 7:**
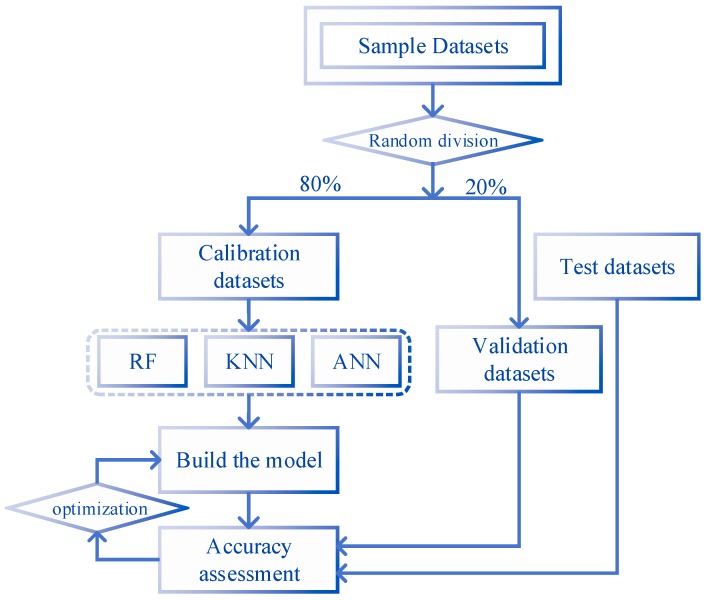
Accuracy assessment scheme.

Accuracy assessment scheme is show in Figure 7. The sample datasets are divided into calibration datasets and validation datasets in this study. Note that there is no overlap between calibration datasets and validation datasets, and they are used for three classifiers of five scenarios with the same location and quantity. Calibration datasets were used to build the model of classifiers, validation datasets were used to optimize the model and verify the classification accuracy, and test datasets were used to output the classification map. We could analyze the classification results by classification accuracy and classification map.

## 3. Results 

### 3.1. Classification Performance of Scenarios

**Figure 8:**
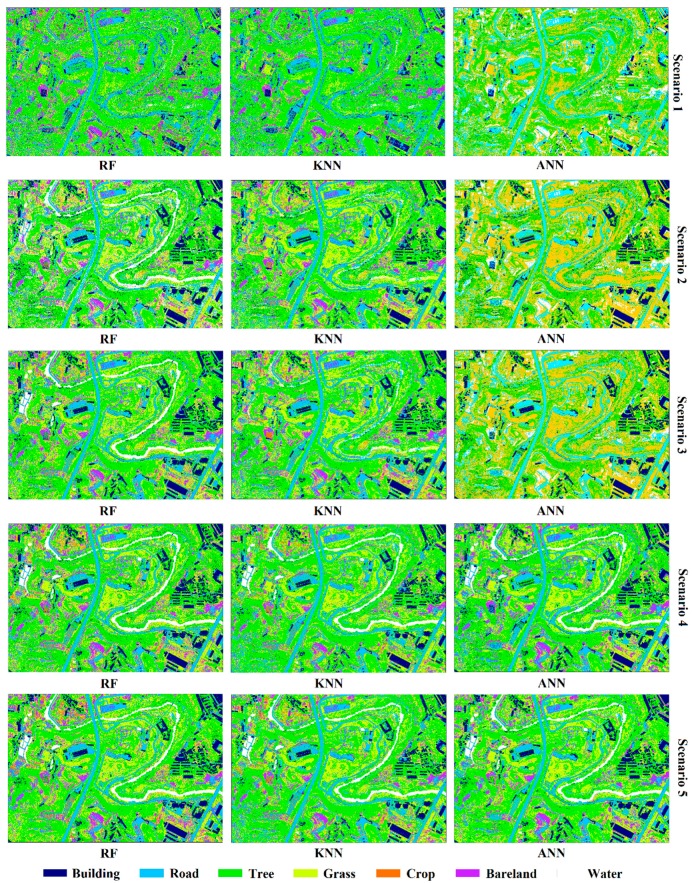
Classification results for five scenarios with three machine learning methods. RF: random forest.

Table 2 summarizes the classification accuracy regarding the five scenarios with different classifiers. As shown in Table 2, the OA and the kappa coefficients of different scenarios changed from 47.18% to 84.75% and 0.31 to 0.80, respectively, which indicate that the classification accuracy significantly depends on the increase/decrease of the input bands as well as the classifiers selection. Note that scenario 5 with RF model performs better than the other models in the suburban land cover classification. It has the highest OA (84.75%) and the highest kappa coefficient (0.80). In addition, land cover discrimination at class level was reflected by UA and PA. Table 3 gives the class level accuracies calculated by CM for each land cover. As shown in Table 3, scenario 5 obtained the relatively maximum land cover discrimination, and each classifier has its own advantage on particular classes. Figure 8 shows the classification results for five scenarios with three machine learning methods. 

As shown in Table 2 and Table 3, the classification performances of each scenario are summarized as follows:

(1) Scenario 1 only used spectral information in the aerial imagery. Most classes were badly misclassified and confused, i.e., buildings and roads, or trees and grasses, because of similar spectral reflectance values, this resulting very low UA and PA by analyzing scenario 1 in Table 3. Additionally, in scenario 1, ANN gave the better performance than the other classifiers (OA = 50.18% and kappa coefficient = 0.35).
(2) When the nDSM was combined with spectral information of aerial imagery (scenario 2), the accuracy of classification significantly improved. The OA and kappa coefficient of the three classifiers were dramatically improved comparing with scenario 1, the lowest OA was 68.75% of KNN which increased 21.57% and the highest was 77.97% of RF which increased 27.16%, the lowest kappa coefficient was 0.59 of KNN which improved 0.28 and the highest was 0.71 of RF which improved 0.36 (Table 2). Correspondingly, the significant decreases of misclassified rate between buildings and roads, and between trees and grasses were observed, where the UA and PA of trees were over 90% (Table 3). However, the distinction between low buildings and trees was not optimistic due to their few high differences.
(3) In order to overcome the confusion between low buildings and trees, scenario 3 contained RBG, nDSM, and RM. In scenario 3, we found that RM plays a key role in distinguishing between the low buildings and trees. In addition, the OA of three classifiers ranged from 70.37% to 81.81%, which was 1.62% higher than the lowest of scenario 2 and 3.84% higher than the highest. The kappa coefficient was from 0.61 to 0.76, which was 0.02 higher than the lowest of scenario 2 and 0.05 higher than the highest (Table 2). The classification accuracies of the seven classes were further improved.
(4) Previous research proved that IM has advantages in material surface differentiation [2,5,12,21,23]. To compare the effects of RM and IM in the suburban classification, we used IM in scenario 4 instead of RM in scenario 3. Few variations in OA and kappa coefficient were observed between scenario 3 and 4. Additionally, crops and waters have higher classification accuracy than scenario 3, roads, barelands, and grasses have the nearly accuracy as scenario 3, while buildings and trees have no higher accuracy than scenario 3 (Table 3).
(5) Considering RM and IM have their own advantages, scenario 5 combined all the variables including RGB, nDSM, RM, and IM. Scenario 5 gave the best performance in classification among all the scenarios. The lowest OA was 72.99% of KNN, the highest was 84.75% of RF with 80.61% of ANN, the lowest kappa coefficient is 0.65 of KNN, and the highest was 0.80 of RF with 0.75 of ANN. The UA and PA of all classes get almost the highest level using RF classifier. However, there were some phenomena indicated that the classification results of KNN and ANN were not stable than RF. For example, in the process of KNN classifier, the PA of bareland was 0.29% lower than that of scenario 3 and 2.15% lower than that of scenario 4, the UA of the buildings was 0.82% lower than scenario 3, while the grasses was 1.39% lower than scenario 4; in the process of ANN classifier, the PA of barelands was 1.12% lower compared with scenario 3 and 5.29% lower compared with scenario 4, the UA of the roads and trees were not performance well than scenario 3 and 4, and the barelands accuracy was not higher than scenario 4.

### 3.2. Performances of Classifiers

**Figure 9:**
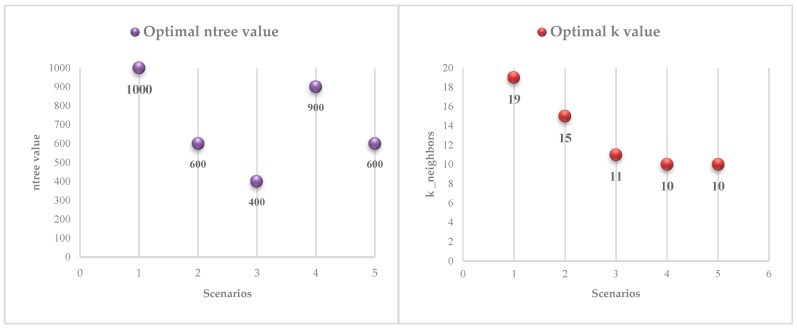
Optimal ntree value of RF (**left**) and optimal k value of KNN in five scenarios (**right**).

**Figure 10:**
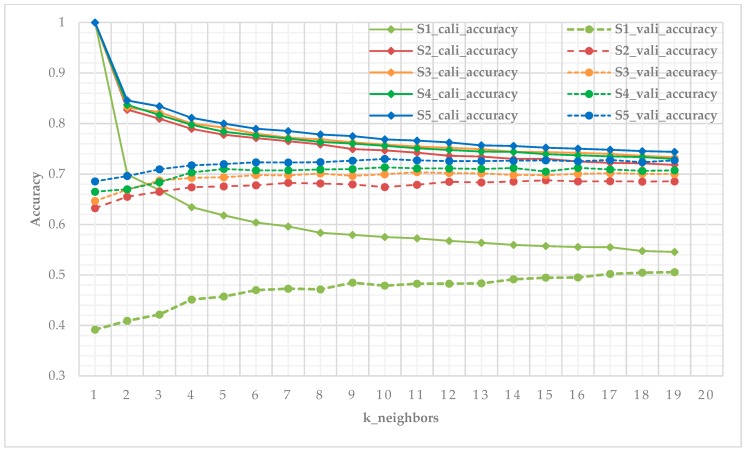
Iterative testing of optimal k value of KNN classifier using calibration data and validation data for five scenarios, we select the n neighbor corresponding to the highest accuracy of validation as the optimal k value (S1_cali_accuracy and S1_vali_accuracy represent the calibration accuracy and validation accuracy of scenario 1, other legends follow this pattern).

Three classifiers were executed with their optimal parameters. In the implementation of RF classifier, the value of ntree was set from 0 to 1000 at an interval of 100 in each scenario to determine the optimal ntree value and produces the best output (Figure 9 left). While implementing the KNN, validation datasets were used to iteratively test (the number of neighbors is between 1 and 20, including 1 but not including 20) the accuracy of the classification model which were constructed by calibration datasets to obtain the optimal k value (Figure 9 right), Figure 10 illustrates the iterative process of optimal k value acquisition of five scenarios. The classifiers were optimized to ensure the highest classification accuracy of each scenario.

**Figure 11:**
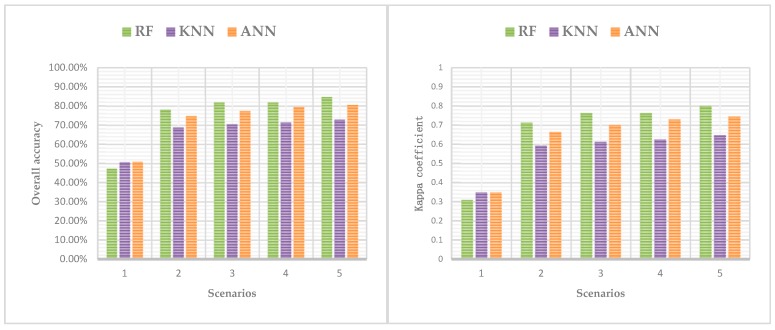
Comparison of the OA and kappa coefficient of RF, KNN, and ANN for five scenarios.

The RF classifier performance was relatively prominent by analyzing and comparing the OA and kappa coefficient of classification results, followed by ANN and KNN (Figure 11). RF achieved the highest accuracy in scenario 2, 3, 4, and 5, except that it was not better than ANN in scenario 1. In particular, the OA of RF in scenario 3 was 0.03% higher than in scenario 4, and the kappa coefficient were both 0.76. However, the OA of KNN in scenario 4 was 0.96% higher than that of scenario 3, the kappa coefficient was 0.02 higher, the OA of ANN was 2.12% higher than scenario 3, and the kappa coefficient was 0.03 higher. These indicated that RM and IM have similar performance in RF classifier (RM is slightly better), but in KNN and ANN, the addition of IM is more helpful to the improvement of classification accuracy than RM, especially in ANN classifier.

### 3.3. Variables Importance Representation

Different variables have different degrees of importance in different datasets, thus, the quantification of the variable importance is not only an important issue of ranking variables before stepwise estimate models, but also to interpret data and understand potential phenomenon in many application problems [30]. To identify the relative variable importance in the models in this study, we used the mean decrease impurity method by summing total impurity reductions at all tree nodes where the variable appears, by comparing the variable importance of five scenarios.

**Figure 12:**
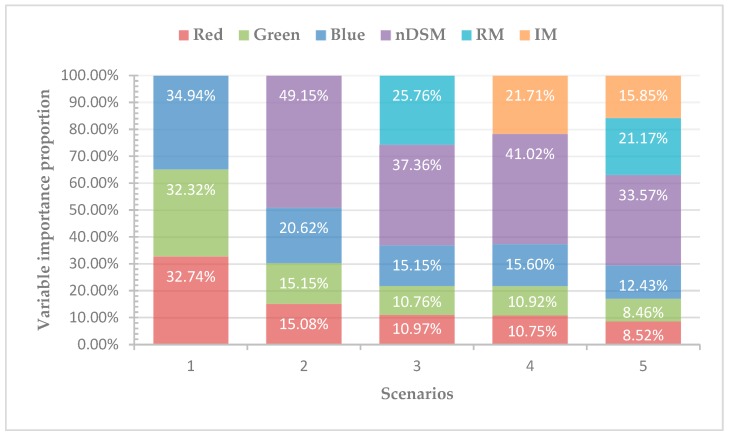
Variable importance from mean decrease impurity method in five scenarios in term of proportion.

We defined the variable importance proportion (VIP) as the proportion of the importance of each variable to the importance of the whole variable in each scenario, where the VIP reflected the contribution for classification of each variable. Figure 12 describes the variable importance by mean decrease impurity method in five scenarios in term of proportion. As shown in Figure 12, in scenario 1, the VIP values of the three bands were similar. Scenario 2 combined RGB with nDSM, it can be seen that nDSM plays a vital role in classification contribution, with the VIP value up to 49.15% far more than the other three bands. In scenario 3, although the VIP of nDSM remained the first, the contribution of RM to classification cannot be ignored. In scenario 4, the VIP value of IM was 21.71%, which was 19.31% lower than that of nDSM. In scenario 5, the order of VIP value is nDSM > RM > IM > Blue > Green > Red.

## 4. Discussion

Previous observational studies on land cover classification concentrations have focused primarily on the urban region [2], such as the city of Fredericton in Canada [5] and the Macheng city in China [13]. However, with the gradual improvement and expansion of urban area to the suburbs, the suburban land cover classification has been ignored to some degree. Additionally, some scholars have examined the satellite or aerial imagery and LiDAR fusion data in land cover classification [3,5,6,7,8]. Many researchers have focused on the introduction of new data source and variables or innovation of classification methods [2,3,14,15,16]. Therefore, the new feature description should be explored. Our research concentrated on the suburban land cover classification and the effects of the new feature on classification results. The exploration of aerial imagery fused with all the LiDAR-derived models using three classifiers (RF, KNN, and ANN) revealed that the fusion of RGB, nDSM, RM, and IM performed best with significant increase in OA and kappa coefficient (Table 2, Table 3), among which RF classifier executed outstanding (OA = 84.75%, kappa coefficient = 0.80).

**Figure 13:**
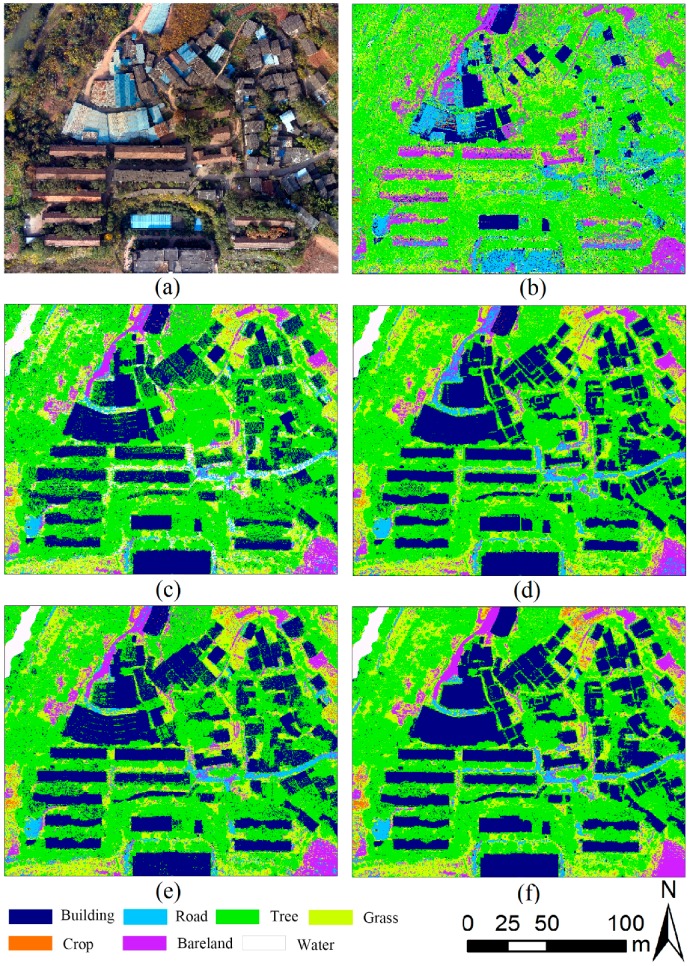
Subgraph of classification results for the five scenarios obtained by using RF classifier: (**a**) the corresponding aerial imagery of the subgraph; (**b**) scenario 1 classification result subgraph with input data of RGB; (**c**) scenario 2 classification result subgraph with input data of RGB and nDSM; (**d**) scenario 3 classification result subgraph with input data of RGB, nDSM, and RM; (**e**) scenario 4 classification result subgraph with input data of RGB, nDSM, and IM; (**f**) scenario 5 classification result subgraph with input data of RGB, nDSM, RM, and IM.

LiDAR-derived models were widely used to enrich the information of imagery. Some researchers have proved that the nDSM and intensity gave the better classification performance in land cover research [2,5,14,22]. However, there are still much useful information about the LiDAR point clouds should be discovered. Given the difference of surface fluctuations degree of ground objects (Figure 3), RM was defined as a new variable for classification using three classifiers. Figure 13 shows subgraph of classification results for the five scenarios obtained by using RF classifier. As shown in Figure 13c–e, visually, RM performed better than nDSM and intensity in distinguishing low buildings from trees in the same height. The low building roofs of Figure 13d was more legible than that in Figure 13c,e, in which indicated that RM can indeed identify the different fluctuations of features. In addition, the classification mapping of scenario 5 obtained the best output (Figure 13f) with the highest OA and kappa coefficient (Table 2). More importantly, the VIP analysis showed that RM contributes more to the classification than IM. These results suggest that RM can effectively improve the classification accuracy of suburban land cover; the role of RM in classification is comparable to that of IM, and it is promising to explore the LiDAR data for the land cover classification.

In a suburban land cover environment, the infrastructure is inadequate and the architectural style is not uniform, such as the cement roads are often mixed with bare soil, the greenland planning is confusing and the residential buildings with different heights, styles and materials. Additionally, some other factors such as the training sample datasets quality, classification methods and the design of classification scheme, affect the quality of classification outcomes [47]. These problems have hindered the accuracy of suburban land cover classification, which is an important reason why the OA accuracy of this paper has not reached more than 90%. In future studies, RM should be applied in different scenes and methods to better test its feasibility in improving classification accuracy. In addition, we should study more available variables to identify the complex ground objects. We should explore more effective algorithms to improve the suburban land cover classification accuracy. 

## 5. Conclusions

In this paper, the aerial imagery and LiDAR-derived models were fused to explore the suburban land cover classification. We divided the study area into seven main land cover classes, i.e., building, road, tree, grass, crop, bareland, and water. All the classes were classified under five scenarios with RF, KNN, and ANN classifiers. The main conclusion of this study are as follows:

(1) The fusion of LiDAR-derived models (i.e., nDSM and IM) and spectral information of the aerial imagery significantly increased the classification accuracy in suburbs, where the landscape is disorderly distributed, the architectural style is not uniform, and the road repair is incomplete.
(2) A new LiDAR-derived model (i.e., RM) was constructed for the classification. RM performs well in improving the accuracy of classification using three classifiers. Additionally, RM has an advantage in distinguishing buildings from trees. Moreover, the contribution of RM to classification is comparable to that of IM.
(3) The fusion of all the variables (i.e., RGB, nDSM, RM, and IM) yielded the best classification results which were estimated by three classifiers, among which RF resulted in the highest classification accuracy (OA = 84.75%, kappa coefficient = 0.80). In addition, the VIP showed that nDSM contributed the most, followed by RM, IM, and spectral information.

In conclusion, we have successfully developed a framework for the fusion of aerial imagery and LiDAR data for the suburban LULC classification. The results and methods could provide meaningful and valuable references for the suburban land cover management and urban–rural integration development.

## Figures and Tables

**Table 1:** Fused datasets input bands used for five combination scenarios.

Scenarios	Datasets	Number of Bands
1	Aerial imagery spectral information(R, G, B)	3
2	Aerial imagery spectral information(R, G, B) + nDSM	4
3	Aerial imagery spectral information(R, G, B) + nDSM + RM	5
4	Aerial imagery spectral information(R, G, B) + nDSM + IM	5
5	Aerial imagery spectral information(R, G, B) + nDSM + RM+ IM	6

**Table 2:** Classification accuracies regarding five scenarios with different classifiers (note: the best overall accuracy (OA) and Kappa Coefficient are shown in **bold**). RF: random forest; KNN: k-nearest neighbor; ANN: artificial neural network.

Scenario	OA (%)			Kappa Coefficient		
	RF	KNN	ANN	RF	KNN	ANN
1	47.18	50.56	50.81	0.31	0.35	0.35
2	77.97	68.75	74.68	0.71	0.59	0.66
3	81.81	70.37	77.26	0.76	0.61	0.70
4	81.78	71.33	79.38	0.76	0.63	0.73
5	**84.75**	72.99	80.61	**0.80**	0.65	0.75

**Table 3:** Class level classification accuracies of three classifiers for each land covers. UA: user’s accuracy; PA: producer’s accuracy; nDSM: normalized digital surface model; RM: surface roughness model; IM: intensity model.

Land Cover	PA (%)			UA (%)		
	RF	KNN	ANN	RF	KNN	ANN
Scenario1: Aerial imageries spectral information(R, G, B)						
Building	51.54	58.46	71.62	27.46	31.15	21.72
Road	49.88	50.87	47.29	59.05	65.46	70.47
Tree	52.51	53.95	54.62	58.09	64.41	68.18
Grass	38.58	43.32	43.23	42.46	45.6	44.1
Crop	41.67	44.44	0	15.96	4.26	0
Bareland	52.38	58.04	58.60	45.29	43.53	49.12
Water	35.62	40.98	0	26.80	25.77	0
Scenario2: Aerial imageries spectral information(R, G, B) + nDSM						
Building	86.01	85.88	84.58	68.03	62.3	69.67
Road	69.65	56.46	64.17	67.13	69.36	64.35
Tree	91.5	89.11	90.26	96.78	78.94	96.56
Grass	70.41	58.6	62.86	82.54	79.15	85.68
Crop	56.52	51.61	0	27.66	17.02	0
Bareland	63.04	64.68	72.85	47.65	44.71	47.35
Water	80.58	48.65	0	85.57	37.11	0
Scenario3: Aerial imageries spectral information(R, G, B) + nDSM + RM						
Building	93.3	91.33	90.29	79.92	64.75	76.23
Road	74.19	59.43	61.27	70.47	70.19	72.70
Tree	94.46	86.30	91.73	98.23	86.59	97.12
Grass	72.21	60.19	68.69	87.81	73.12	86.81
Crop	71.05	26.47	50	28.72	19.15	2.13
Bareland	66.39	64.17	73.19	47.06	47.94	50.59
Water	100	52	0	100	40.21	0
Scenario4: Aerial imageries spectral information(R, G, B) + nDSM + IM						
Building	90.14	91.12	90.63	78.69	63.11	71.31
Road	74.26	60.83	67.09	69.92	69.64	73.82
Tree	93.89	82.12	90.75	97.12	83.48	96.78
Grass	73.25	60.61	69.85	87.06	75.38	84.42
Crop	75.51	78.38	0	39.36	30.85	0
Bareland	66.41	66.03	77.27	50	40.59	50
Water	100	96.97	93.07	100	98.97	96.91
Scenario5: Aerial imageries spectral information(R, G, B) + nDSM + RM+ IM						
Building	94.17	94.55	94.04	86.07	63.93	84.02
Road	79.22	61.72	69.75	73.26	71.87	71.31
Tree	95.77	85.53	92.16	98	87.14	96.45
Grass	76.52	61.35	70.81	90.08	73.99	85.93
Crop	77.14	84.09	57.14	57.45	39.36	4.26
Bareland	70	63.88	71.98	51.47	42.65	49.12
Water	100	96.97	98.98	100	98.97	100
